# Disease-Ligand Identification Based on Flexible Neural Tree

**DOI:** 10.3389/fmicb.2022.912145

**Published:** 2022-06-06

**Authors:** Bin Yang, Wenzheng Bao, Baitong Chen

**Affiliations:** ^1^School of Information Science and Engineering, Zaozhuang University, Zaozhuang, China; ^2^School of Information and Electrical Engineering, Xuzhou University of Technology, Xuzhou, China; ^3^Xuzhou No.1 People’s Hospital, Xuzhou, China

**Keywords:** virtual screening, network pharmacology, flexible neural tree, grammar-guided genetic programming, salp swarm algorithm

## Abstract

In order to screen the disease-related compounds of a traditional Chinese medicine prescription in network pharmacology research accurately, a new virtual screening method based on flexible neural tree (FNT) model, hybrid evolutionary method and negative sample selection algorithm is proposed. A novel hybrid evolutionary algorithm based on the Grammar-guided genetic programming and salp swarm algorithm is proposed to infer the optimal FNT. According to hypertension, diabetes, and Corona Virus Disease 2019, disease-related compounds are collected from the up-to-date literatures. The unrelated compounds are chosen by negative sample selection algorithm. ECFP6, MACCS, Macrocycle, and RDKit are utilized to numerically characterize the chemical structure of each compound collected, respectively. The experiment results show that our proposed method performs better than classical classifiers [Support Vector Machine (SVM), random forest (RF), AdaBoost, decision tree (DT), Gradient Boosting Decision Tree (GBDT), KNN, logic regression (LR), and Naive Bayes (NB)], up-to-date classifier (gcForest), and deep learning method (forgeNet) in terms of AUC, ROC, TPR, FPR, Precision, Specificity, and F1. MACCS method is suitable for the maximum number of classifiers. All methods perform poorly with ECFP6 molecular descriptor.

## Introduction

Computer-aided drug design (CADD) has gradually become an indispensable emerging technology in the research and development of a new drug (Leelananda and Steffen, 2016; Tong et al., 2019; Maia et al., 2020). CADD technology reduces the capital, time, and labor cost of drug development and greatly improves the efficiency of the research and development of new drug (Gomeni et al., 2001). Virtual screening is one of the important comprehensive technical means in CADD, which is a process of discovering new ligands on the basis of biological structure based on the computer methods (Guasch et al., 2016; Olubiyi et al., 2020; Rajguru et al., 2020). It is a new technology and method for innovative drug research. By using the high-speed computing of computer, a small number of potential active compounds are screened from a large number of candidate compounds, so as to greatly reduce the blindness of subsequent experimental verification. In the future, virtual screening technology will become an important means to explore the relevant biochemical space because of its many advantages, such as high efficiency, high speed, low cost, and so on (Zaslavskiy et al., 2019; Guo et al., 2021; Maddah et al., 2021; Selvaraj et al., 2021; Yang et al., 2021).

In the past decade, virtual screening has been applied to the medical and the pharmaceutical researches widely (Meng et al., 2011; Bajusz et al., 2017). The most commonly used virtual screening method is molecular docking, and the software involved contains AutoDock, SLIDE, DOCK, Flex X, etc. (Morris et al., 1996; Kellenberger et al., 2004; Taufer et al., 2005). Fischer et al. (2021) utilized virtual screening method to screen 25, 56, 750 compounds in order to make the analysis about the binding of small molecules to translationally controlled tumor protein. Baxter et al. (2000) utilized molecular docking to screen ligand-receptor complexes in virtual database and tabu search method was utilized to assist this work. Talluri (2021) utilized Vina and SMINA to make molecular docking to predict potential drugs for the treatment of Corona Virus Disease 2019 (COVID-19). Zhou et al. (2016) screened the compounds of Chicory, which were bundled with concentrated nucleoside transporter 2 (CNT2) in order to validate that CNT2 as the potential target of chicory could reduce the absorption of purine nucleosides in the intestine. Meenakumari et al. (2019) made docking analysis between 17 coumarin derivatives and carbonic anhydrase IX (CAIX) to screen the ligands. Thiyagarajan et al. (2016) made molecular docking between the 3D structures of focal adhesion kinase and S6 kinase and 60 natural compounds to obtain the new specific inhibitors, and the findings could provide help for the treatment of tumorigenesis and metastasis.

In order to improve the time and accuracy of virtual screening, some machine learning methods have been utilized to assist or replace molecular docking (Berishvili et al., 2018; Zaki et al., 2021). Wang et al. (2016) proposed a new virtual screening based on ensemble learning and SVM to tackle with protein-ligand in action fingerprint. Zhang Y. et al. (2019) investigated the performances of 8 classifiers containing decision tree (DT), KNN, SVM, random forest (RF), extremely randomizer tree, AdaBoost, gradient boosting tree, and XGBoost with ACC inhibitor data for the researches of drug design and discovery. Zhang et al. (2017) proposed a new scoring function based on machine learning to screen the compounds targeting the viral neuraminidase protein so as to make anti-influenza therapy. Chen et al. (2011) proposed a ligand screening algorithm based SVM to discovery lead compounds. Bustamam et al. (2021) proposed a dipeptidyl peptidase-4 (DPP-4) inhibitors identification method based on Rotation Forest and Deep Neural Network with the fingerprint datasets for the treatment of type 2 diabetes mellitus. Zheng et al. (2020) utilized Naïve Bayesian and recursive partitioning to select the important active chemical components from many compounds in Xiaoshuan Tongluo formula with ECFP_6 and MACCS feature sets for treating stroke.

Virtual screening of disease-related compounds can narrow the scope of analysis in network pharmacology research. In this paper a new virtual screening method based on flexible neural tree (FNT) model is proposed to screen the disease-related active compounds. A novel hybrid evolutionary algorithm based on Grammar-guided genetic programming and salp swarm algorithm is proposed to infer the structure and parameters in each FNT model. The 3 diseases (hypertension, diabetes, and COVID-19) related compounds are searched from the up-to-date literatures. The unrelated compounds are selected by negative sample selection algorithm from DUD-E website. About 4 kinds of molecular descriptors (ECFP6, MACCS, Macrocycle, and RDKit) are utilized to numerically characterize the chemical structures of related and unrelated compounds of diseases, respectively. We make the investigation about the performances of these 4 molecular descriptors.

## Materials and Methods

### Flexible Neural Tree Model

**FIGURE 1:**
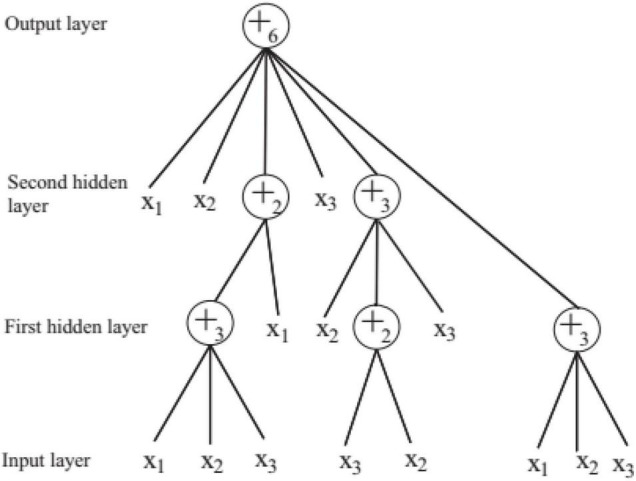
An example of flexible neural tree.

In order to solve the automatic design problem of artificial neural network, FNT was proposed, which is a hierarchical, multilayer, and irregular artificial neural network (Chen et al., 2012). FNT can transform a single and fixed neural network model into a special tree model that can change flexibly between various levels. It could overcome the difficulty of structural optimization of common neural network, have strong adaptive ability for various classification and prediction problems, and obtain high classification and prediction accuracy. In this paper, FNT is proposed to predict active disease-related compounds. An example of structure of FNT model is showed in Figure 1. AFNT includes input layer, several hidden layers and output layer. The nodes in the input layer are created randomly from terminal set *T* = {*x*_1_, *x*_2_,…,*x*_*n*_}. The nodes in the hidden layers are selected randomly from terminal set and operator set *F* = { + _2_, + _3_,…, + _*n*_}. The output layer contains one node.

**FIGURE 2:**
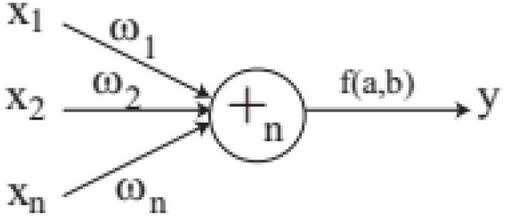
A flexible neuron operator.

In FNT, each layer is randomly generated according to the operation set and terminal set. The maximum depth of tree is set in advance. If an operator instruction + _*n*_ is selected, *n* branches are created randomly from set *T* and *F*, which are terminal variables and operators. And *n* weights are generated randomly. If a terminal variable is selected, the corresponding branch is terminated. When FNT is created randomly, the depth of FNT could not exceed the maximum depth. + _*n*_ is depicted in Figure 2 and is calculated as follows.


(1) $$n\,e\,t_{n}=\sum_{j=1}^{n}w_{j}\,x_{j}.$$

The final output of + _*n*_ is calculated by activation function, which is given as follows.


(2) $$y=f\,\left(n\,e\,t_{n},a_{n},b_{n}\right)=e^{-{\left(\frac{n\,e\,t_{n}-a_{n}}{b_{n}}\right)}^{2}}.$$

Where *a*_*n*_ and *b*_*n*_ are parameters of activation function.

### Model Optimization Algorithm

#### Grammar-Guided Genetic Programming

Grammar-guided genetic programming (GGGP) was proposed in order to overcome the shortcomings of genetic programming (Wu and Chen, 2007). In this paper, GGGP is utilized to search the optimal structure of FNT model. In GGGP, context-free grammar (CFG) model is utilized to guide the evolutionary process of GP in order to search the optimal solution faster.

The CFG model contains a quadruple, which is represented as *G* = {*N, T, P*,∑}, where *N* is non-terminal symbol set, *T* is terminal symbol set, *P* is production rule set and ∑ is beginning symbol set. The 4 sets satisfy the conditions: *N*⋂*T* = ϕ and ∑ ∈ *N*. An element in production rule set is represented as *x*→*y*, where *x* ∈ *N*, and *y* ∈ *N*⋃*T*. Assuming that terminal set and operator set are set as *T* = {*x*_1_, *x*_2_,…,*x*_*n*_}, and *F* = { + _2_, + _3_}, 4 sets of CFG model are defined:*N* = {*s, exp, var, op*2, *op*3}, *T* = { + _2_, + _3_, *x*_1_, *x*_2_, …, *x*_*n*_}, ∑ = {*s*}, and *P* is represented with Eq. (3) or Eq. (4).


(3) $$\begin{array}{c} s\to \exp\\ \exp\to \exp o\,p\,2\,\exp\\ \exp\to o\,p\,3\,\exp\exp\exp\\ \exp\to v\,a\,r\\ o\,p\,2\to {+}_{2}\\ o\,p\,3\to {+}_{3}\\ v\,a\,r\to x_{1}\,\left|x_{2}\right|\,\ldots |x_{n} \end{array}$$

(4) $$\begin{array}{c} s\to \exp\\ \exp\to o\,p\,2\,\exp\exp\\ \exp\to o\,p\,3\,\exp\exp\exp\\ \exp\to v\,a\,r\\ o\,p\,2\to {+}_{2}\\ o\,p\,3\to {+}_{3}\\ v\,a\,r\to x_{1}\,\left|x_{2}\right|\,\ldots |x_{n} \end{array}$$

Generate the initial population randomly. When generating each individual tree, the non-terminal node *S* is started with. Then the subtree of each non-terminal node is derived in top-down and left-right order according to the rules of the syntax model. When all non-terminal nodes in the tree have sub-trees, stop the derivation process of the tree, and then judge the depth of the tree. If the depth is greater than the predefined maximum depth, the tree is considered invalid, and a tree is regenerated after deletion. If the depth is less than the maximum depth, the tree is considered and can be saved to the population. Then 3 genetic operators (replication, crossover, and mutation) are utilized to generate a new population in the iteration process.

#### Salp Swarm Algorithm

The Salp swarm algorithm (SSA) is a new swarm optimization algorithm proposed by Mirjalili et al. (2017). The main idea of SSA comes from simulating the group behavior of salp chain (Babaei et al., 2020; Ren et al., 2021). In this algorithm, salp chain is divided into 2 groups: leader and follower. The leader is at the head of the salp chain, and the followers are at the back of the chain. In each iteration, the leader directs the followers to move in a chain toward the food. In the process of moving, the leader makes global search, while the follower makes full local search, which greatly avoid falling into local optimization. The leader’s leadership role for the followers behind will be weaker and weaker. The followers behind will not blindly move toward the leader, which could maintain the diversity of the population. Therefore, this movement mode makes the salp chain have a strong ability of global search and local development. Because of its simple implementation, fast convergence speed, and easy computer implementation, SSA is utilized to optimize the parameters of FNT model. The SSA is given as follows in detailed.

(1) Initialize the population. Suppose that population size is *m*, the dimension is *n*, the upper bound of the search space is $X_{\max}=\left\{X_{\max}^{1},X_{\max}^{2},\ldots ,X_{\max}^{n}\right\}$, the lower bound is $X_{\min}=\left\{X_{\min}^{1},X_{\min}^{2},\ldots ,X_{\min}^{n}\right\}$. The positions of salp population are created randomly by the following equation.


(5) $$X_{i}=r\,a\,n\,d\,\left(\right)\times \left(X_{\max}-X_{\min}\right)+X_{\min}.$$

(2) Give the fitness values of population according to the fitness function defined in advanced. In the iteration process, the position of the food is not clear, so the fitness values of all individual salps are calculated and sorted. And the position of salp with the optimal fitness value is set as the current food position, which is set as *F* = {*F*^1^, *F*^2^,…, *F_n_*}.

(3) Positions of leader and followers are updated. The leader is responsible for searching food to lead the moving direction of the whole group. The position of the leader is updated as follows (Chen and Mu, 2021).


(6) $$X_{1}^{i}=\left\{ \begin{array}{c} F^{i}+c_{1}\times \left(\left(X_{\max}^{i}-X_{\min}^{i}\right)\times c_{2}+X_{\min}^{i}\right)\,c_{3}\geq 0.5,\\ F^{i}-c_{1}\times \left(\left(X_{\max}^{i}-X_{\min}^{i}\right)\times c_{2}+X_{\min}^{i}\right)\,c_{3}<0.5. \end{array}\right.$$

Where $X_{1}^{i}$ and *F^i^* are the *i*-th positions of leader (the first salp) and food. *c*_2_ and *c*_3_ are random number. *c*_1_ is the convergence factor in SSA, which could play the role of balancing global search and local development. *c*_1_ is calculated as follows.


(7) $$c_{1}=2\,e^{-{\left(\frac{4\,t}{T}\right)}^{2}}.$$

Where *t* is the current generation and *T* is the maximum generation.

The positions of the followers are updated according to Newton’s laws of motion, which is defined as follows.


(8) $$X_{i}^{j}=0.5\times a\,t^{2}+v_{0}\,t.$$

(9) $$\begin{array}{c} a=\frac{v_{f\,i\,n\,a\,l}-v_{0}}{△\,t},\\ v_{f\,i\,n\,a\,l}=\frac{X_{i}^{j}-X_{i}^{j-1}}{△\,t}. \end{array}$$

Where *a* is acceleration. The difference between two adjacent iterations is 1 and *v*_0_ = 0, so Eq. (8) could be defined as follows.


(10) Xij′=Xij-Xij-12.

(4) Update the fitness values of new population and the position of food. If the end condition is satisfied, algorithm is stopped; otherwise go to step (3).

### Screen Disease-Related Compounds by Our Proposed Method

**FIGURE 3:**
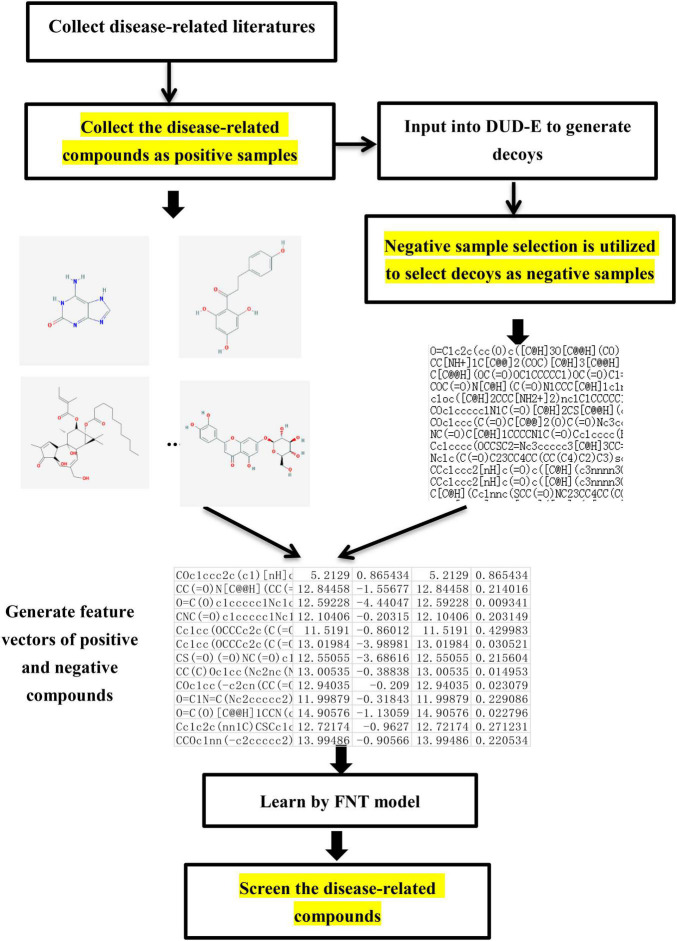
The flowchart of screening disease-related compounds algorithm.

Virtual screening is needed in the research of network pharmacology to select the disease-related compounds. In this paper, a novel virtual screening method based on FNT, hybrid evolutionary method and negative sample selection algorithm is proposed, which is depicted in Figure 3.

(1) Disease-related compound dataset collection. Search the up-to-date literatures for treating diseases according to the name of disease. By consulting these literatures with data mining method, the active compounds for the treatment of the disease are collected as the positive compound samples. In order to generate the unrelated compounds, the positive compounds are input into DUD-E database to generate the corresponding decoys, which are set as negative samples (Mysinger et al., 2012). There are too many decoys generated compared to the number of positive samples. In order to balance the proportion of positive samples and negative samples, negative sample selection based on Tanimoto index (Algorithm 1) is presented to choose a certain number of decoys that are quite different from the positive sample set. Tanimoto index could measure the distance between the 2 compounds, which can measure the similarity between 2 sets (Klekota et al., 2005), which can solve the relationship between 0 and 1 well. The greater Tanimoto index is, the higher the similarity of 2 sets is. The Tanimoto index of 2 sets *A* and *B* is calculated as followed.


(11) $$T\,\left(A,B\right)=\frac{A\cap B}{A\cup B}.$$

**Algorithm 1:** Negative sample selection algorithm.

**Input:** disease-related compound set [*c*_1_, *c*_2_,…,*c*_*m*_] (*m* is the number of compounds),
the generated decoy set [*g*_1_, *g*_2_,…,*g*_*n*_] (*n* is the number of decoys)
**Output:** the selection negative compound set [*n*_1_, *n*_2_,…,*n*_2*m*_]
for *i* = 1;*i*≤*n*;*i* + + do
*sum*_*i*_ = 0;
for *j* = 1;*j*≤*m*;*j* + + do
*T*_*ij*_ = *Tanimotoindex*(*g*_*i*_, *c*_*j*_);
*sum*_*i*_ = *sum*_*i*_ + *T*_*ij*_;
End
End
Sort the decoy set according to [*sum*_1_, *sum*_2_,…,*sum*_*n*_];
Select the decoys with 2*m* smallest Tanimoto indexes as negative compound set;

(2) Screening process. The related and unrelated molecules collected are all chemical structures. To facilitate the compounds collected inputting into flexible neural tree model, 4 kinds of molecular descriptors (ECFP6, MACCS, Macrocycle, and RDKit) are utilized to numerically characterize the chemical structure of each compound (Todeschini and Consonni, 2009). ECFP6 contains 2,048 features, which denotes all possible molecular routes retrieved from the atom according to radius 3 and each bit denotes whether the special stator structure exists. MACCS contains 166 molecular characteristic sites, such as ISOTOPE, ATOMIC NO, 4M RING, and GROUP VIII. Macrocycle contains 1,613 features, which refer the information about the ring-size, sugars, and ester functional groups. RDK it contains 208 features, such as number of valence electros, number of radical electrons, charge information, and number of Aliphatic Carbocycles. Cross-validation method is utilized to divide the training and testing datasets to test the performance of our proposed method. With the feature vector of each compound in the training dataset as the input, flexible neural tree model is utilized to train with the feature datasets. A hybrid evolutionary method based on grammar-guided genetic programming and salp swarm algorithm is proposed to search the optimal structure and parameters of FNT model. For the unknown compounds of testing dataset, the feature vectors are used as the input of the optimal FNT model to obtain the output results. If the result is higher than 0.5, the compound is identified to be disease-related; otherwise, it is unrelated.

## Experiment Results and Discussion

In order to test the effectiveness of our method, the important compounds were collected, which were involved in the treatment of hypertension, diabetes, and COVID-19. The related compounds of these 3 diseases are regarded as positive samples and the numbers of samples are 67, 124, and 88, respectively. Negative sample selection method is utilized to select the inactive compounds about hypertension, diabetes and COVID-19, and the numbers of negative samples are 134, 248, and 176, respectively. The 4 kinds of molecular descriptors (ECFP6, MACCS, Macrocycle, and RDKit) are utilized to numerically characterize related and unrelated compounds of diseases, respectively.

**FIGURE 4:**
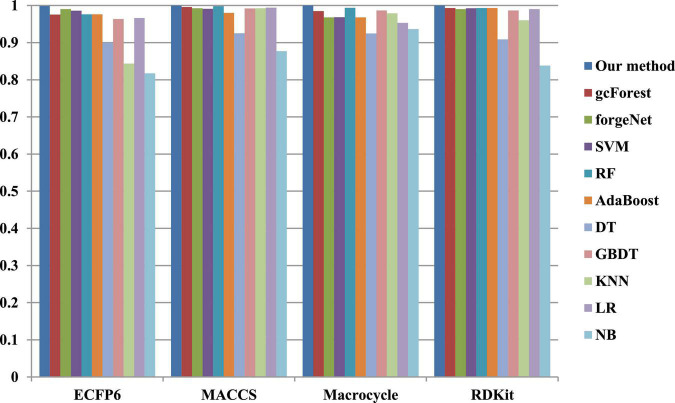
AUC performances of 11 methods with hypertension dataset.

**FIGURE 5:**
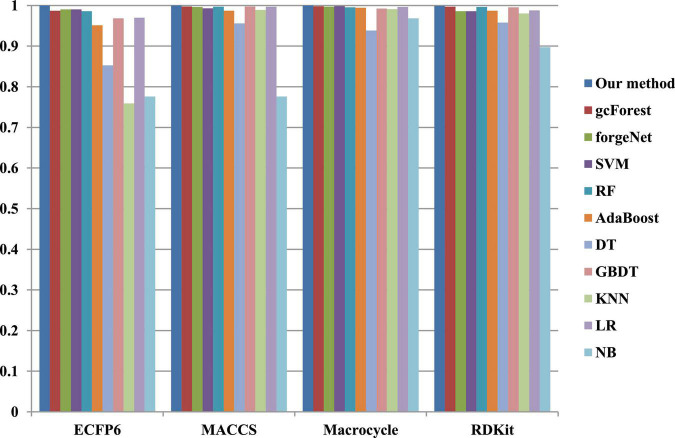
AUC performances of 11 methods with diabetes dataset.

**FIGURE 6:**
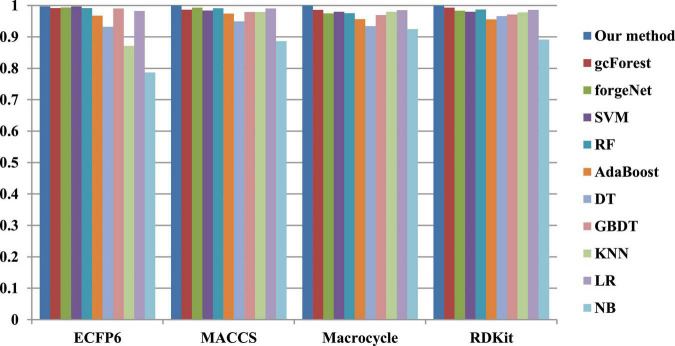
AUC performances of 11 methods with COVID-19 dataset.

The 10-cross validation method is utilized to test the performance of our method. SVM (Hearst et al., 1998), RF (Breiman, 2001), AdaBoost (Collins et al., 2002), decision tree (DT) (Safavian and Landgrebe, 1991), GBDT (Zhang B. et al., 2019), KNN, logical regression (LR) (Collins et al., 2002), gc Forest (Zhou and Feng, 2017), forgeNet (Kong and Yu, 2020), and Naive Bayes (NB) (Kim et al., 2006)are also utilized to identify disease-related compounds of three diseases. In our method, operator set is set as *F* = { + _2_, + _3_, + _4_, + _5_}, population size is set as 30 and the maximum depth of tree is set as 5. In SVM, linear kernel function is selected. In RF, the number of trees is set as 100. In GBDT, the number of regression trees is set as 200. In DT, CART algorithm is utilized. The parameters of other algorithms are set by default. The AUC performances of 11 methods with the datasets about hypertension, diabetes, and COVID-19 are shown in Figures 4–6, respectively. From Figure 4, it could be seen that with ECFP6, Macrocycle, and RDKit methods, our method has the highest AUC performances among 11 methods. With MACCS method, the AUC values obtained by our method and RF are very close to 1.0, which are 0.999889 and 0.997772, respectively. For Figure 5, in terms of AUC, it could be clearly seen that our method performs best with ECFP6, MACCS, and RDKit methods. With Macrocycle feature method, our method, gcForest, and SVM could obtain the better AUC values than other 8 methods, which are 1, 0.99803, and 0.998435, respectively. By the comparison of these 3 methods, our method performs best, which show that our method is a good classifier for disease-compound identification problem. For Figure 6, with ECFP6molecular descriptor, our method and SVM could obtain the higher AUC values than other 9 methods, which are 0.996901 and 0.99703. With other molecular descriptors, our method could obtain the better performances, which are equal to or very close to 1.0.

TPR, FPR, Precision, Specificity, and F1 are also utilized to test the performances of 11 methods for compound identification about 3 diseases. TPR denotes the ratio of true disease-related compounds identified against all true disease-related ones. FPR denotes the ratio of disease-related compounds identified erroneously against all true disease-unrelated ones. Precision denotes the ratio of true disease-related compounds identified against all disease-related ones identified. Specificity is the ratio of true disease-unrelated compounds identified against all true disease-unrelated ones. F1 could evaluate a classifier comprehensively with Precision and Recall. TPR, FPR, Precision, Specificity, and F1performances of11 methods with the datasets about hypertension, diabetes and COVID-19 are listed in Tables 1–3, respectively. In Table 1, with ECFP6 method, our method has the highest TPR performance among 11 classifiers, which shows that our method could identify more true disease-related compounds. In terms of FPR, Precision and Specificity, forgeNet and RF perform best, which reveal that all the true disease-unrelated compounds are identified. But our method could obtain the highest F1 performance. Overall our method could obtain the more accurate identification results. With MACCS, Macrocycle, and RDKit, our method could obtain the best performances of TPR, FPR, Precision, Specificity, and F1.

**TABLE 1:** Prediction performances of 11 methods with hypertension dataset.

Molecular descriptors	Methods	TPR	FPR	Precision	Specificity	F1
ECFP6	Our method	**0.985075**	0.022222	0.956522	0.977778	**0.970588**
	gcForest	0.955224	0.155556	0.752941	0.844444	0.842105
	forgeNet	0.895522	**0**	**1**	**1**	0.944882
	SVM	0.880597	0.007407	0.983333	0.992593	0.929134
	RF	0.880597	**0**	**1**	**1**	0.936508
	AdaBoost	0.835821	0.037037	0.918033	0.962963	0.875
	DT	0.835821	0.044444	0.903226	0.955556	0.868217
	GBDT	0.850746	0.051852	0.890625	0.948148	0.870229
	KNN	0.686567	**0**	**1**	**1**	0.814159
	LR	0.970149	0.311111	0.607477	0.688889	0.747126
	NB	0.731343	0.096296	0.790323	0.903704	0.75969
MACCS	Our method	**1**	**0.007407**	**0.985294**	**0.992593**	**0.992593**
	gcForest	0.970149	0.051852	0.902778	0.948148	0.935252
	forgeNet	0.925373	0.018587	0.96124	0.981413	0.942966
	SVM	0.940299	0.02963	0.940299	0.97037	0.940299
	RF	0.940299	0.014815	0.969231	0.985185	0.954545
	AdaBoost	0.895522	0.044444	0.909091	0.955556	0.902256
	DT	0.895522	0.051852	0.895522	0.948148	0.895522
	GBDT	0.925373	0.014815	0.96875	0.985185	0.946565
	KNN	0.925373	0.02963	0.939394	0.97037	0.932331
	LR	0.970149	0.066667	0.878378	0.933333	0.921986
	NB	0.940299	0.192593	0.707865	0.807407	0.807692
Macrocycle	Our method	**0.984375**	**0**	**1**	**1**	**0.992126**
	gcForest	0.9375	0.09009	0.857143	0.90991	0.895522
	forgeNet	0.921875	0.018018	0.967213	0.981982	0.944
	SVM	0.890625	0.027027	0.95	0.972973	0.919355
	RF	0.90625	0.027027	0.95082	0.972973	0.928
	AdaBoost	0.953125	0.027027	0.953125	0.972973	0.953125
	DT	0.921875	0.072072	0.880597	0.927928	0.900763
	GBDT	0.90625	0.036036	0.935484	0.963964	0.920635
	KNN	0.921875	0.072072	0.880597	0.927928	0.900763
	LR	0.9375	0.153153	0.779221	0.846847	0.851064
	NB	0.9375	0.09009	0.857143	0.90991	0.895522
RDKit	Our method	**0.985075**	**0**	**1**	**1**	**0.992481**
	gcForest	0.955224	0.02963	0.941176	0.97037	0.948148
	forgeNet	0.895522	0.022222	0.952381	0.977778	0.923077
	SVM	0.940299	0.014815	0.969231	0.985185	0.954545
	RF	0.865672	0.014815	0.966667	0.985185	0.913386
	AdaBoost	0.925373	0.014815	0.96875	0.985185	0.946565
	DT	0.873134	0.055762	0.886364	0.944238	0.879699
	GBDT	0.895522	0.02963	0.9375	0.97037	0.916031
	KNN	0.865672	0.044444	0.90625	0.955556	0.885496
	LR	0.955224	0.02963	0.941176	0.97037	0.948148
	NB	0.895522	0.214815	0.674157	0.785185	0.769231

*Bold values denote the best performances.*

**TABLE 2:** Prediction performances of 11 methods with diabetes dataset.

Molecular descriptors	Methods	TPR	FPR	Precision	Specificity	F1
ECFP6	Our method	0.991935	0.012048	0.97619	0.987952	**0.984**
	gcForest	0.967742	0.124498	0.794702	0.875502	0.872727
	forgeNet	0.916031	**0.007605**	**0.983607**	**0.992395**	0.948617
	SVM	0.935484	0.02008	0.958678	0.97992	0.946939
	RF	0.862903	0.008032	0.981651	0.991968	0.918455
	AdaBoost	0.879032	0.036145	0.923729	0.963855	0.900826
	DT	0.806452	0.100402	0.8	0.899598	0.803213
	GBDT	0.854839	0.02008	0.954955	0.97992	0.902128
	KNN	**1**	0.939759	0.346369	0.060241	0.514523
	LR	0.967742	0.15261	0.759494	0.84739	0.851064
	NB	0.604839	0.052209	0.852273	0.947791	0.707547
MACCS	Our method	**0.975806**	**0**	**1**	**1**	**0.987755**
	gcForest	**0.975806**	0.02008	0.960317	0.97992	0.968
	forgeNet	0.951613	0.024096	0.951613	0.975904	0.951613
	SVM	0.935484	0.024096	0.95082	0.975904	0.943089
	RF	0.943548	0.012048	0.975	0.987952	0.959016
	AdaBoost	0.943548	0.032129	0.936	0.967871	0.939759
	DT	0.951613	0.040161	0.921875	0.959839	0.936508
	GBDT	0.975806	0.02008	0.960317	0.97992	0.968
	KNN	0.951613	0.044177	0.914729	0.955823	0.932806
	LR	0.975806	0.02008	0.960317	0.97992	0.968
	NB	0.967742	0.417671	0.535714	0.582329	0.689655
Macrocycle	Our method	**0.991453**	**0**	**1**	**1**	**0.995708**
	gcForest	0.982906	0.028037	0.950413	0.971963	0.966387
	forgeNet	0.957265	0.009346	0.982456	0.990654	0.969697
	SVM	0.974359	0.018692	0.966102	0.981308	0.970213
	RF	0.957265	0.014019	0.973913	0.985981	0.965517
	AdaBoost	0.957265	0.018692	0.965517	0.981308	0.961373
	DT	0.91453	0.037383	0.930435	0.962617	0.922414
	GBDT	0.965812	0.046729	0.918699	0.953271	0.941667
	KNN	0.923077	0.018692	0.964286	0.981308	0.943231
	LR	0.982906	0.042056	0.927419	0.957944	0.954357
	NB	0.974359	0.042056	0.926829	0.957944	0.95
RDKit	Our method	0.959677	**0**	**1**	**1**	**0.979424**
	gcForest	0.959677	0.02008	0.959677	0.97992	0.959677
	forgeNet	**0.967742**	0.012048	0.97561	0.987952	0.97166
	SVM	0.951613	0.008032	0.983333	0.991968	0.967213
	RF	0.935484	0.012048	0.97479	0.987952	0.954733
	AdaBoost	0.943548	0.016064	0.966942	0.983936	0.955102
	DT	0.943548	0.028112	0.943548	0.971888	0.943548
	GBDT	0.943548	0.008032	0.983193	0.991968	0.962963
	KNN	0.903226	0.012048	0.973913	0.987952	0.937238
	LR	0.959677	0.024096	0.952	0.975904	0.955823
	NB	0.951613	0.204819	0.698225	0.795181	0.805461

**TABLE 3:** Prediction performances of 11 methods with COVID-19 dataset.

Molecular descriptors	Methods	TPR	FPR	Precision	Specificity	F1
ECFP6	Our method	**0.965909**	**0**	**1**	**1**	**0.982659**
	gcForest	**0.965909**	0.101695	0.825243	0.898305	0.890052
	forgeNet	0.931818	0.00565	0.987952	0.99435	0.959064
	SVM	0.920455	0.011299	0.975904	0.988701	0.947368
	RF	0.931818	**0**	**1**	**1**	0.964706
	AdaBoost	0.896226	0.025882	0.945274	0.974118	0.920097
	DT	0.909091	0.045198	0.909091	0.954802	0.909091
	GBDT	0.886364	0.028249	0.939759	0.971751	0.912281
	KNN	0.897727	0.435028	0.50641	0.564972	0.647541
	LR	0.988636	0.214689	0.696	0.785311	0.816901
	NB	0.636364	0.062147	0.835821	0.937853	0.722581
MACCS	Our method	**1**	**0**	**1**	**1**	**1**
	gcForest	0.954545	0.011299	0.976744	0.988701	0.965517
	forgeNet	0.943182	0.008499	0.982249	0.991501	0.962319
	SVM	0.931818	0.011299	0.97619	0.988701	0.953488
	RF	0.954545	**0**	**1**	**1**	0.976744
	AdaBoost	0.886364	0.016949	0.962963	0.983051	0.923077
	DT	0.931818	0.033898	0.931818	0.966102	0.931818
	GBDT	0.931818	0.00565	0.987952	0.99435	0.959064
	KNN	0.954545	0.028249	0.94382	0.971751	0.949153
	LR	0.954545	0.016949	0.965517	0.983051	0.96
	NB	0.863636	0.090395	0.826087	0.909605	0.844444
Macrocycle	Our method	**0.965517**	**0**	**1**	**1**	**0.982456**
	gcForest	0.954023	0.006536	0.988095	0.993464	0.97076
	forgeNet	0.954023	**0**	**1**	**1**	0.976471
	SVM	0.942529	0.006536	0.987952	0.993464	0.964706
	RF	0.942529	0.006536	0.987952	0.993464	0.964706
	AdaBoost	0.954023	**0**	**1**	**1**	0.976471
	DT	0.908046	0.039216	0.929412	0.960784	0.918605
	GBDT	0.896552	0.03268	0.939759	0.96732	0.917647
	KNN	0.931034	0.019608	0.964286	0.980392	0.947368
	LR	0.954023	0.026144	0.954023	0.973856	0.954023
	NB	0.885057	0.039216	0.927711	0.960784	0.905882
RDKit	Our method	**0.965909**	**0**	**1**	**1**	**0.982659**
	gcForest	0.943182	0.022599	0.954023	0.977401	0.948571
	forgeNet	0.943182	0.011299	0.976471	0.988701	0.959538
	SVM	0.943182	0.011299	0.976471	0.988701	0.959538
	RF	0.931818	0.00565	0.987952	0.99435	0.959064
	AdaBoost	0.931818	0.016949	0.964706	0.983051	0.947977
	DT	0.943182	0.011299	0.976471	0.988701	0.959538
	GBDT	0.943182	0.011299	0.976471	0.988701	0.959538
	KNN	0.954545	0.016949	0.965517	0.983051	0.96
	LR	0.943182	0.028249	0.943182	0.971751	0.943182
	NB	0.897727	0.112994	0.79798	0.887006	0.84492

*Bold values denote the best performances.*

In Table 2, with ECFP6 method, KNN has the highest TPR performance among 11 classifiers, which is 1.0. The result shows that KNN could identify all true disease-related compounds. In terms of FPR, Precision, and Specificity, forgeNet perform better than other 10 methods. But our method could also obtain the highest F1 performance. Overall our method could obtain the more accurate identification results. With MACCS and Macrocycle, our method could obtain the best performances of TPR, FPR, Precision, Specificity, and F1. With RDKit, our method performs best in terms of FPR, Precision, Specificity, and F1, while forgeNet could obtain the best TPR performance. For Table 3, our method performs best with 4 kinds of molecular descriptors in terms of 5 criterions. All results show that our method could predict disease-related compounds more accurately than gcForest, forgeNet, SVM, RF, AdaBoost, DT, GBDT, KNN, LR, and NB.

According to the performances of 11 methods with the datasets from 3 diseases and 4 molecular descriptors, 11 methods are ranked. For each molecular descriptor, the averaged ranking results of each method are listed in Table 4. From Table 4, we can see that our method, gcforest, forgenet, RF, GDBT, and LR perform best with MACCS feature set, while SVM and DT perform best with RDKit feature set. AdaBoost, KNN and NB perform better with Mordred feature set than the other 3 feature sets. All methods perform poorly with ECFP6 molecular descriptor. The results also show that the different molecular descriptors of compounds are suitable for the different classifiers and the ranking results can provide the guidance for each classifier to choose the appropriate molecular descriptor to solve the problem in the future. On the whole, MACCS method is suitable for the maximum number of classifiers. In future research, MACCS method can be preferred for a new classifier.

**TABLE 4:** Averaged ranking scores of 11 methods with 3 datasets.

	ECFP6	MACCS	Macrocycle	RDKit
Our method	3.33	**1.67**	2	2.67
gcForest	3.67	**1.83**	2.33	2.17
forgeNet	2.5	**2.17**	2.33	3
SVM	2.83	2.5	2.5	**2.17**
RF	2.83	**1.33**	2.5	3.17
AdaBoost	3.5	2.5	**1.83**	2.17
DT	4	1.83	2.5	**1.67**
GBDT	3.5	**1.33**	2.83	2.33
KNN	4	1.83	**1.67**	2.5
LR	3.83	**1.17**	2.83	2.17
NB	3.67	2.83	**1**	2.33

**FIGURE 7:**
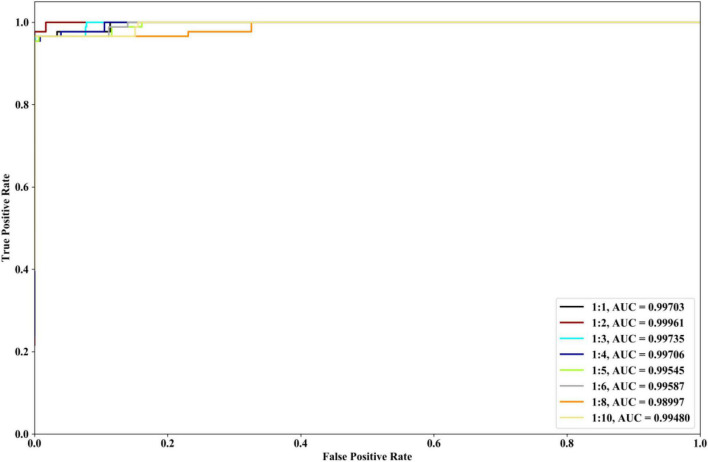
Performances of our method with COVID-19 dataset and the different ratios of positive and negative samples.

We investigate the performances of our method with different ratios of positive and negative samples. The 8 kinds of ratios (1:1, 1:2, 1:3, 1:4, 1:5, 1:6, 1:8, and 1:10) are selected and COVID-19 dataset is utilized. The identification results are depicted in Figure 7. From Figure 7, it could be seen that when the ratios are 1:1, 1:2, 1:3, and 1:4, our method could have the better ROC and AUC performances. The excessive imbalance of data may affect the classification performance of the algorithm.

## Conclusion

In order to sort the candidate compounds in a traditional Chinese medicine prescription and narrow the scope of analysis in network pharmacology research accurately, this paper proposes a new virtual screening method based on flexible neural tree (FNT) model, hybrid evolutionary method, and negative sample selection algorithm to screen the disease-related active compounds. 3 diseases (hypertension, diabetes, and Corona Virus Disease 2019) related compounds are collected from the up-to-date literatures. The unrelated compounds are selected by negative sample selection algorithm from DUD-E website. 4 kinds of molecular descriptors (ECFP6, MACCS, Macrocycle, and RDKit) are utilized to characterize the features of related and unrelated compounds of diseases, respectively. The experiment results show that our proposed method performs better than classical classifiers (SVM, RF, AdaBoost, DT, GBDT, KNN, LR, and NB), up-to-date classifier (gcForest) and deep learning method (forgeNet) in terms of AUC, ROC, TPR, FPR, Precision, Specificity, and F1.

We also investigate the performances of 11 methods with 4 kinds of molecular descriptors. The results show that our method, gcforest, forgenet, RF, GDBT, and LR perform best with MACCS feature set, while SVM and DT perform best with RDKit feature set, AdaBoost, KNN and NB perform best with Mordred feature set. With ECFP6 molecular descriptor all methods perform poorly.

In the paper, our proposed method has been successfully applied to hypertension, diabetes, and Corona Virus Disease. In the future, our method will be utilized to identify other chronic disorders related compounds, such as cancers, coronary heart disease, and rheumatoid disease.

## Data Availability Statement

The original contributions presented in this study are included in the article/supplementary material, further inquiries can be directed to the corresponding author/s.

## Author Contributions

WB conceived the method and wrote the main manuscript text. BY designed the method and conducted the experiments. All authors reviewed the manuscript.

## Conflict of Interest

The authors declare that the research was conducted in the absence of any commercial or financial relationships that could be construed as a potential conflict of interest.

## Publisher’s Note

All claims expressed in this article are solely those of the authors and do not necessarily represent those of their affiliated organizations, or those of the publisher, the editors and the reviewers. Any product that may be evaluated in this article, or claim that may be made by its manufacturer, is not guaranteed or endorsed by the publisher.
